# CIRSE Position Statement on Endovascular Aortic Repair

**DOI:** 10.1007/s00270-025-04201-9

**Published:** 2025-10-15

**Authors:** Mohamad Hamady, Catharina S. P. van Rijswijk, Hicham Kobeiter, Maria Antonella Ruffino, Fabrizio Fanelli, Patrick Haage, Romaric Loffroy, Stefan Müller-Hülsbeck, Gerard O’Sullivan, Florian Wolf, Robert A. Morgan

**Affiliations:** 1 Imperial College, St Mary’s Campus, London, UK; 2 Department of Radiology, C2-S, Leiden University Medical Center, Leiden, The Netherlands; 3 Radiology Department, H. Mondor Hospital, Assistance Publique-Hôpitaux de Paris, University Paris Est Creteil, Creteil, France; 4 Interventional Radiology, Institute of Imaging of Southern Switzerland—EOC Lugano, Lugano, Switzerland; 5 Vascular and Interventional Radiology Department, “Careggi” University Hospital—University of Florence, Florence, Italy; 6 Helios University Hospital, University Witten/Herdecke, Wuppertal, Germany; 7 Department of Diagnostic and Interventional Radiology, François-Mitterrand University Hospital, Dijon, France; 8 Academic Hospital Christian-Albrechts-University Kiel, Kiel, Germany; 9 University Hospital Galway, Galway, Ireland; 10 Division of Cardiovascular and Interventional Radiology, Medical University of Vienna, Vienna, Austria; 11 St George’s University of London, London, UK

## Introduction

Since the introduction of standard endovascular aortic repair for infrarenal aortic aneurysms (EVAR) and complex fenestrated (FEVAR) and branched stent grafts (BEVAR) for thoracoabdominal and thoracic aortic repairs pathology, interventional radiologists (IRs) have performed important roles in these aortic therapies, including device development, technical advances, and the generation of evidence [1–4]. Pre-eminent interventional radiologists, particularly in Europe, have led or co-authored landmark publications and clinical trials involving endovascular repair [5–7].

However, as evidence in support of standard and complex EVAR/ TEVAR has accrued, and an increasing number of vascular surgeons, interventional cardiologists, and cardiothoracic surgeons have embraced these techniques, a definable shift has begun to occur among the operators dominating the field. This transformation is primarily influenced by reimbursement systems, classical referral patterns, political territorial issues, and certain inherent aspects within the practice of interventional radiology. The relative scarcity of interventional radiologists in specific countries, the rapid expansion of the range of procedures performed by IRs outside the aorta, and relatively insufficient clinical presence of IRs are among the contributing factors that have led to the marginalisation of interventional radiologists from aortic procedures in some countries. Even when interventional radiologists participate in procedures at specific centres, the profiles generated in these centres do not accurately reflect their true roles.

Recently, CIRSE conducted a survey of its members to assess the current role of IRs in aortic practice. The survey clearly demonstrated that IRs continue to play a significant role in standard and complex EVAR as part of multidisciplinary teams or even as the primary operators. The survey also identified specific challenges and highlighted potential areas for improvement and support for IRs in endovascular aortic repair, including training and expanding the workforce.

### Why IRs are Important in Aortic Interventions

Although EVAR is commonly perceived as less invasive and potentially simpler than traditional open repair, the truth is that a safe and successful endovascular procedure requires considerable attention to planning and technical expertise for safe deployment and endovascular problem solving. Interventional radiologists bring their expertise in imaging, catheter and guidewire skills, and dexterity in device manipulations, and a minimally invasive philosophy to improve the technical outcomes of EVAR and complex EVAR and TEVAR. Optimal outcomes of standard and complex endovascular repair are best achieved by teams of IRs and vascular surgeons who bring different but complementary skills to procedures to provide the best outcomes for patients [8].

### Radiation Protection and Radiation Doses

Radiation exposure is relatively high in aortic procedures, especially in complex cases. The effects of radiation, both stochastic and non-stochastic, should not be underestimated. Interventional radiologists, with their background in radiation safety training, can be an asset during EVAR procedures to assist in reducing radiation exposure to patients and operators alike. A recent report from the UK investigated radiation exposures during endovascular aortic repairs in centres where interventional radiologists and vascular surgeons collaborate, revealing notable findings in record keeping as well as levels of exposure [9]. In comparison with a recent US study where surgeons solely performed endovascular aortic repairs, the UK study found patients had at least a 40% reduction in radiation exposure, and overall procedure times were significantly shorter [10]. Although the UK study did not specifically examine personnel doses, it is reasonable to infer that radiation doses to staff would also be significantly lower.

It has long been recognised that aortic interventions should be performed using the highest imaging quality, not only to provide the best patient care but also to reduce radiation doses to patients and operators alike [11]. The vast majority of IR angiography units are equipped with state-of-the-art machinery, offering an excellent collaborative environment in the most cost-effective way, avoiding duplication of services and unnecessary expenditure. This is a point that should be emphasised to hospital management.

### How IR can Improve Care and Contribute to the Future Health Practices

IRs play a crucial role in post-aortic interventions imaging follow-up, helping to diagnose issues when the aneurysm sac enlarges and managing endoleaks. The number of endovascular procedures continues to increase, with the expectation that this trend will continue in the coming years due to technological and device advances, the gradual move away from open surgery and the consequent inevitable decline in open surgical skills. Interventional radiology departments are strategically well positioned to collaborate with surgical colleagues to provide comprehensive patient care. More efforts must be made by IRs and national societies to persuade health authorities that collaborative initiatives yield superior patient care and are more cost-effective by optimising hospital resources without duplicating facilities or incurring additional costs. It is intuitive that a multidisciplinary approach optimises clinical care and avoids inappropriate clinical practices [12]. Interventional radiologists, wherever they work, should strive to be involved in these discussions and demonstrate to both colleagues and hospital management the essential contributions that interventional radiology can offer endovascular aortic repair and to overcome the barrier to clinical ownership. True clinical interventional radiology requires outpatient clinics, ward rounds, inpatient beds, and longitudinal follow-up.

### How IR of the Future Should be Prepared

For IRs to be integral to vascular care or to regain their role in some countries, several prerequisites are necessary. Advancing clinical skills and device knowledge are essential for both current and future practices of IR in general, especially in the vascular field. More time and focus for building and maintaining procedural IR cases with less diagnostic duties is required. Engagement in research, presence in aortic multidisciplinary teams, and positioning of IRs at the forefront of technological advancements are crucial for enhancing clinical care. Furthermore, one of the painful lessons from the history of IR is the cost the speciality has paid due to a lack of visibility. National societies, supported by CIRSE, should continually highlight the significant roles IR plays in standard and complex EVAR. Finally, an ongoing commitment by national and international IR societies to training in endovascular aortic procedures for IRs is also essential.

In conclusion, as was demonstrated in the recent CIRSE survey, IRs continue to play a vital role in providing aortic interventions across Europe. Collaboration with other surgical specialities such as vascular surgery and cardiothoracic surgery ensures optimal clinical care for aortic patients. Societies should work together to promote good medical practice and to prioritise patient interests.
